# H-ferritin nanoparticle-mediated antibody delivery across the blood-brain barrier

**DOI:** 10.3389/fnagi.2026.1839772

**Published:** 2026-06-03

**Authors:** Ziwei Yuan, Laura Rué, Tom Jaspers, Marie-Lynn Cuypers, Maarten Dewilde

**Affiliations:** 1 Laboratory for Therapeutic and Diagnostic Antibodies, KU Leuven – University of Leuven, Leuven, Belgium; 2 PharmAbs, The KU Leuven Antibody Center, KU Leuven – University of Leuven, Leuven, Belgium

**Keywords:** antibody delivery, BACE1, blood-brain barrier, H-ferritin, transferrin receptor 1

## Abstract

**Background:**

Therapeutic monoclonal antibodies (mAbs) show great promise for treating neurological disorders thanks to their high selectivity and potency. However, their clinical potential is significantly limited by poor penetration across the blood-brain barrier (BBB). It has been shown that antibodies targeting transferrin receptor 1 (TfR1) can reach the brain after peripheral administration. In addition, human H-ferritin (HFn) naturally undergoes TfR1-mediated transcytosis, making it a compelling alternative candidate platform for brain-directed drug delivery. This study evaluates the feasibility of using HFn as a generic shuttle to transport therapeutic antibodies into the brain.

**Methods:**

An anti-BACE1 monoclonal antibody (1A11) was chemically conjugated to HFn, and the resulting nanoparticles (1A11-HFn NPs) were assessed *in vitro* and *in vivo* for their structural integrity, target binding, BBB permeability, BACE1 inhibition, and pharmacological effects. Hereto, HFn and the mAb 1A11 were produced in *Escherichia coli* and CHO cells, respectively, followed by purification of each protein. Conjugation of 1A11 to HFn was achieved using the heterobifunctional linker NHS-PEG-Mal, after which 1A11-HFn NPs were isolated. Binding to human BACE1 and human TfR1 was confirmed. Structural characterization was performed via transmission electron microscopy (TEM). Pharmacokinetics (PK) and pharmacodynamics (PD) were examined in humanized apical domain TfR1 knock-in (hApiTfrc KI) mice after intravenous (IV) administration, with evaluation of both plasma and brain tissue.

**Results:**

1A11-HFn NPs were successfully generated with confirmed dual binding to hBACE1 and hTfR1. TEM imaging verified the structural integrity of the nanoparticles. *In vivo* studies demonstrated increased brain levels of 1A11-HFn NPs at days 1 and 3 post-administration, though levels declined below the limit of detection (LOD) by day 7. The amount reaching the brain at day 1 but not day 3 or 7 was sufficient to inhibit BACE1 activity, indicating a pharmacological meaningful dose could reach the brain after peripheral administration.

**Conclusion:**

These findings demonstrate that HFn can serve as an effective shuttle to deliver mAbs across the BBB at pharmacologically relevant concentrations. This work provides foundational evidence supporting HFn-based nanoparticles as a promising platform for brain-targeted delivery of biological therapeutics.

## 1 Introduction

In recent years, monoclonal antibody (mAb) therapies have become increasingly predominant in the pharmaceutical market due to their superior efficacy and safety (Lu et al., 2020). Currently, approximately 93% of all approved mAb therapies come from four major regulatory regions: US, Europe, Japan, and China. The number of approvals in these regions continues to rise annually, highlighting a decade of strong, steady growth in the global mAb development (Lyu et al., 2022). However, the majority of mAb therapies target cancers, immune-related disorders, infectious diseases, and hematological conditions. In contrast, only about 3% of therapeutic antibodies target central nervous system (CNS) diseases, primarily due to the blood-brain barrier (BBB), which severely limits the brain penetration of large molecules (Pardridge, 2019; Lyu et al., 2022). Physiologically, the BBB is formed by specialized endothelial cells, tight junctions, astrocytes, and other supporting components that separate the brain parenchyma from the systemic circulation (Alahmari, 2021). Although this barrier is essential for maintaining homeostasis and preventing pathogen entry, it poses a major challenge for the delivery of macromolecular therapeutics (Zhao et al., 2022).

To overcome this physical blockade, researchers are exploiting endogenous transport pathways for drug transport. This endogenous transport across the BBB can occur through several pathways, including passive paracellular and transcellular diffusion, carrier-mediated transport (CMT), receptor-mediated transcytosis (RMT), and adsorptive-mediated transcytosis (AMT) (Rathi et al., 2022). Among these mechanisms, passive diffusion is generally restricted to small, lipid-soluble molecules. When it comes to larger molecules, such as biological therapeutics like antibodies and enzymes, the most promising strategy to cross the BBB is based on RMT (Preston et al., 2014). RMT is initiated when affinity binders, fused to a drug cargo such as mAbs, engage specific endogenous receptors on the luminal membrane of brain microvascular endothelial cells (BMECs). This binding triggers receptor-mediated endocytosis, intracellular trafficking, and vesicular sorting, culminating in the fusion of vesicles with the abluminal membrane and the release of drug fusion molecules into the brain parenchyma (Terstappen et al., 2021).

To harness these pathways, mAbs can be engineered to engage specific BBB receptors or transporters, such as the transferrin receptor (TfR), Insulin-like growth factor 1 receptor (IGF1R), insulin receptor (IR), basigin (CD147), the glucose transporter (GLUT1), or the CD98hc subunit of the LAT1 transporter (Yu et al., 2014; Boado et al., 2016; Zuchero et al., 2016; Chew et al., 2023; An et al., 2025). These receptor-targeted strategies have emerged as leading approaches for enabling efficient and selective delivery of therapeutic antibodies into the brain. Notably, TfR-based strategies have achieved significant clinical milestones. For example, Izcargo^®^ (JCR Pharmaceuticals), an anti-TfR-idursulfase conjugate, has been approved in Japan in 2021 for clinical use to treat Hunter syndrome (Giugliani et al., 2021). Other TfR targeting shuttles are under clinical evaluation, e.g., Roche’s trontinemab, a bispecific antibody fusing an amyloid-beta (Aβ) binding domain with a TfR1 “Brain shuttle™” module, is currently in Phase 3 clinical trials for Alzheimer’s disease (NCT07170150).

Nanoparticles (NPs), defined as units ranging from 1 to 100 nm in diameter, offer an alternative strategy for brain drug delivery (Masserini, 2013). They are playing an increasingly important role in precision medicine and are becoming advanced platforms for therapeutic delivery (Mitchell et al., 2021). NPs can enhance the stability, solubility, and bioavailability of therapeutic agents while enabling targeted transport across complex biological barriers, including cellular membranes and the BBB (Mitchell et al., 2021). A key feature of many NPs is their core-shell architecture, which facilitates the encapsulation of a wide range of therapeutic payloads, including small-molecule drugs, nucleic acids, and proteins (Wang et al., 2006; Farokhzad and Langer, 2009; Shi et al., 2017; Tetter and Hilvert, 2017). Furthermore, surface functionalization with targeting ligands or antibodies enables selective delivery to specific tissues or cell types (Torchilin, 2014). These features have led to broad applications of NPs in drug delivery, imaging, and gene therapy (Farokhzad and Langer, 2009; Torchilin, 2014; Patra et al., 2018). In particular, their ability to protect cargo and modulate pharmacokinetics (through precise control over size, shape, surface charge, chemistry, and responsiveness) makes them highly attractive for addressing biological barriers (Mitragotri et al., 2014; Blanco et al., 2015). While various BBB-crossing NPs exist, including liposomal formulations, polymers, dendrimers, and metallic NPs, these are predominantly utilized for chemotherapy (Hersh et al., 2022). Among these platforms, protein-based NPs such as ferritin have recently attracted attention as promising and biologically compatible nanocarriers for drug delivery. Ferritin is a ubiquitously expressed natural iron storage protein that maintains iron homeostasis by releasing iron from its core within lysosomes through a controlled process known as ferritinophagy (Mancias et al., 2014; Koralewski et al., 2018). Wild-type ferritin can form a self-assembly 24-subunits cage-like protein structure, which is composed of two types of polypeptide chains: H (Heavy) and L (Light) chains (Adelman et al., 1975). Each subunit consists of four α-helices forming a bundle and a fifth short C-terminal α-helix (Honarmand Ebrahimi et al., 2015). The resulting nanoparticle features a shell thickness of 2 nm and an internal cavity of 8 nm, which can be utilized to encapsulate various therapeutic molecules (Liang et al., 2014). Besides its potential to host a cargo, it possesses an inherent affinity for TfR1, which is highly expressed on BMECs and the most targeted receptor in the development of RMT-based brain delivery approaches (Li et al., 2010). Endogenously, ferritin exhibits BBB-traversing ability via RMT through TfR1 (Fan et al., 2018; Wang et al., 2022). It binds to an epitope located on the apical domain of TfR1 (Montemiglio et al., 2019), which is spatially distinct from the helical and protease-like domains recognized by endogenous transferrin (Cheng et al., 2004). As a result, ferritin-based NPs are only minimally affected by competition with circulating transferrin under physiological conditions (Li et al., 2010; Montemiglio et al., 2019). Furthermore, the 24-subunit architecture of the ferritin nanoparticle enables multivalent interactions with TfR1, leading to enhanced binding avidity. Combined with the elevated expression of TfR1 in tumor cells, which has been reported to be up to 100-fold higher than in normal tissues (Fan et al., 2012), this property significantly strengthens the potential of ferritin-based NPs as effective platforms for CNS-targeted tumor delivery (Zhang et al., 2026). Notably, the heavy chain ferritin (HFn) subunit exhibits a significantly higher binding affinity for TfR1 than either the light chain (LFn) or wild-type ferritin. In addition, HFn demonstrates superior binding to human TfR1 (hTfR1) compared to mouse TfR1 (mTfR1), making it the preferred chain to generate NPs for brain-targeted therapies (Li et al., 2010). When expressed recombinantly, both LFn and HFn form 24-subunits cage-like protein structures, just like wild-type ferritin. Functionally, HFn-based NPs have demonstrated effective delivery of therapeutic agents across the BBB. For example, HFn has been used to transport small molecules into the brain parenchyma, e.g., encapsulated paclitaxel can traverse the BBB and effectively kill glioma cells (Liu et al., 2020). Beyond small molecule delivery, researchers have developed HFn-based nanocarriers fused to antibody(-fragments) to retarget ferritin NPs, e.g., toward cancer-associated fibroblasts (Sitia et al., 2021). HFn has also demonstrated the ability to mediate antibody transport across *in vitro* BBB models (Rizzuto et al., 2021). More recently, HFn-enabled brain delivery of the mAb trastuzumab has been reported, inhibiting breast cancer brain metastases *in vivo*, although further studies are required to fully validate and optimize this promising platform (Sevieri et al., 2023).

**FIGURE 1:**
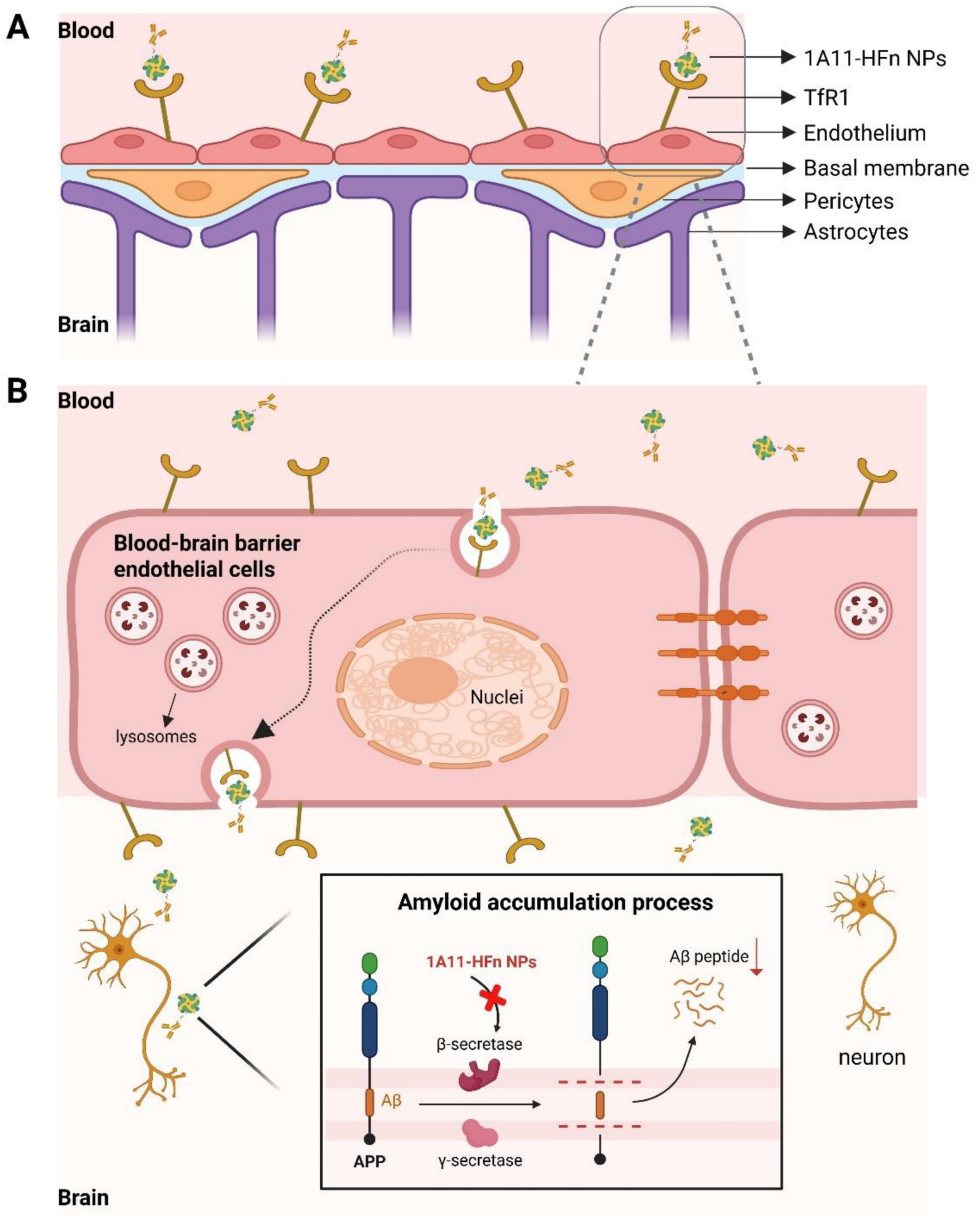
Schematic illustration of 1A11-HFn nanoparticles (NPs) employing a dual-targeting strategy for β-secretase 1 (BACE1) inhibition. It can cross the blood-brain barrier (BBB) via TfR1-mediated transcytosis. After entering the brain parenchyma, the antibody component binds strongly to BACE1 on neurons, resulting in a reduction of Aβ peptides. **(A)** Schematic representation of the blood–brain barrier. **(B)** Intracellular trafficking of 1A11-HFn NPs and inhibition mechanism of amyloid-β generation.

In this study, we investigated whether the inherent BBB-crossing capacity of HFn NPs can be used to shuttle antibodies into the brain at therapeutically relevant concentrations (Figure 1). To validate the delivery capacity of HFn for this project, we selected β-secretase 1 (BACE1) inhibition as a model target. BACE1 is the primary *in vivo* enzyme responsible for initiating the formation of Aβ peptides, which are generated through the sequential cleavage of amyloid precursor protein (APP) by β-secretase 1 and γ-secretase (Lin et al., 2000; Hampel et al., 2021). We conjugated an anti-BACE1 mAb (1A11) to HFn and after *in vitro* characterization, evaluated whether the complex could effectively lower Aβ concentrations in the brain after peripheral administration *in vivo*, providing evidence for successful BBB-crossing. To increase the translational potential, we’ve worked with human and not mouse HFn, and validated them in humanized apical domain TfR1 knock-in (hApiTfrc KI) mice.

## 2 Materials and methods

### 2.1 Cloning, expression, and purification of HFn

The human FTH1 cDNA (HG13217-G, Sino Biological Inc, Beijing, China), encoding HFn, was used as the template for amplification of the HFn coding sequence by polymerase chain reaction (PCR) by using the forward primer 5’-GGAATTCCATATGACGACCGCGTCCA-3’ and the reverse primer 5’-CGGGATCCTTAGCTTTCATTATCACTGTCTCC-3’. These primers introduced NdeI and BamHI restriction sites flanking the start and stop codons, respectively. The double digested PCR product was then ligated into NdeI/BamHI digested plasmid pET-30a(+) under catalysis of T4 DNA ligase and the ligation mixture was transformed into competent *Escherichia coli* DH5α cells following standard procedures. The resulting pET-30a(+)/HFn plasmid, screened by appropriate restriction digests and verified by DNA sequencing, was used to transform into the expression strain *E. coli* BL21 (DE3). Bacteria were cultured in 1 L of Luria Broth (LB) supplemented with 50 mg/L kanamycin at 37 °C. When the optical density at 600 nm (OD_600_) reached 0.6–0.8, protein expression was induced with 1 mM isopropyl β-D-1-thiogalactopyranoside (IPTG), followed by overnight incubation at 28 °C. After incubation, *E. coli* cells were harvested by centrifugation at 5,000 *g* for 45 min and the pellets were resuspended in phosphate buffered saline (PBS) buffer (50 mM PO_4_^3–^, 0.15 M NaCl, pH 7.4) with cOmplete™ protease inhibitor cocktail EDTA-free (Roche Diagnostics, Mannheim, Germany), and DNase (Sigma, Darmstadt, Germany; 20 μg/mL as final concentration). Bacteria were first lysed by repeatedly pipetting the suspension up and down using a 25 mL serological pipette, secondly by using a high-pressure homogenizer (Avestin, Duxford, UK) (10,000–20,000 psi) until the cell lysates were clear, following the centrifugation of lysates at 10,000 *g* for 30 min. After removing the cell debris, the resulting supernatants were heated at 65 °C for 20 min and the final supernatants containing HFn were stored at −20 °C until purification.

The resulting supernatant was precipitated by ammonium sulfate (520 g/L), and the precipitate was collected by centrifugation at 22,000 *g* for 45 min. The pellet was then dissolved into PBS. After removal of ammonium sulfate by dialysis, the resulting supernatant was further purified by size exclusion chromatography (SEC) using a HiLoad™ 16/600 Superose™ 6 pg preparative SEC column (Cytiva, Marblorough, United States) followed by a HiLoad™ 16/600 Superdex™ 200 pg column (Cytiva). The concentration of HFn was determined by the BCA protein assay kit (Thermo Fisher Scientific, Waltham, United States) using BSA as the standard.

### 2.2 Preparation of mAb 1A11

The anti-BACE1 mAb 1A11 was composed of human variable regions (VL and VH) fused to a human IgG1 Fc domain and a human kappa constant region. In addition, the Fc domain contains the LALA-PG mutations (L234A, L235A, P329G), which abolish effector function by preventing Fcγ-receptor and complement binding. Plasmid synthesis, expression in Chinese Hamster Ovary (CHO) cells, and subsequent purification were performed by GenScript (Piscataway, NJ, United States).

### 2.3 Endotoxin removal of HFn

TritonTM X-114 solution (TX-114; Merck, Darmstadt, Germany; 648468) was added to the protein solution to a final TX-114 concentration of 2% v/v. The solution was incubated at 4°C for 30 min. Subsequently, the sample was transferred to a water bath set at 37°C and incubated for 10 min followed by centrifugation at 20,000 *g* for 20 min at 37°C. The upper part containing the protein was separated from the TX-114 layer by means of pipetting. The extraction procedure was repeated seven times. Bio-Beads SM-2 (Bio-Rad, Hercules, United States; 1528920) with high affinity for Triton were added to the collected sample and incubated overnight at 4°C under constant stirring. Bio-Beads were removed by sedimentation or centrifugation. The detergent-free, endotoxin-reduced protein samples were collected for subsequent SEC purification.

### 2.4 Endotoxin measurement of HFn

Endotoxin levels in the purified HFn protein were quantified using the Endosafe^®^ nexgen-PTS™ portable endotoxin testing system (Charles River Laboratories, Wilmington, MA, United States), following the manufacturer’s instructions. The final endotoxin concentration of the HFn sample was determined to be < 5.00 EU/mL.

### 2.5 1 A11-HFn conjugation reaction

Amine-containing 1A11 mAbs and sulfhydryl-containing HFn were covalently conjugated by a heterobifunctional crosslinker with N-hydroxysuccinimide (NHS) ester and maleimide (Mal) groups (Malhex-NH-PEG-O-C_3_H_6_-CONHS, 5 kDa, Rapp Polymere Gmbh, Tubingen, Germany). The conjugation consisted of two steps. First, mAbs were reacted with a 20-fold molar excess of the crosslinker, added dropwise in PBS (pH 7.5), and incubated at the room temperature (RT) on a rotator mixer for 30 min. The unreacted linker was removed by multiple wash cycles with PBS (pH 7.0) using 30 kDa Amicon centrifugal devices (MilliporeSigma, Darmstadt, Germany), during which the mAb concentration was maintained between 3 and 5 mg/mL. In the second step, HFn was added dropwise at a final HFn:mAb molar ratio of 1:1 in PBS (pH 7.0) and incubated overnight at 4 °C on a rotator mixer. Large aggregates were removed by centrifugation (12,000 *g*, 30 min, 4 °C), followed by filtration through 0.2 μm filters and SEC purification using a Superose 6 gel-filtration column (Cytiva) equilibrated with PBS. The eluted fractions were then characterized by sodium dodecyl sulfate–polyacrylamide gel electrophoresis (SDS-PAGE) and dynamic light scattering (DLS).

### 2.6 Characterization of HFn and 1A11-HFn NPs

The hydrodynamic diameters of the purified HFn, mAb 1A11, and 1A11-HFn were determined using DLS on an Uncle device (Unchained Labs, United States). In addition, the same samples were analyzed by SDS-PAGE. Protein concentrations were quantified spectrophotometrically by measuring the absorbance at 562 nm using a BCA protein assay kit (Thermo Fisher Scientific) according to the manufacturer’s instructions.

### 2.7 TEM of HFn and 1A11-HFn NPs

The morphology of the HFn and 1A11-HFn NPs was determined by transmission electron microscopy (TEM) using a JEM-1400Flash Electron Microscope (Jeol, Tokyo, Japan). Briefly, samples were prepared by placing 5 μL sample (∼150 μg/mL) onto Formvar-coated 100-mesh copper grids for 5 min. The excess liquid was blotted, and the grids were subsequently stained by applying a drop of 1% aqueous uranyl acetate solution for 1 min. The solution was removed by blotting, and the grids were allowed to dry. Imaging was performed at an acceleration voltage of 80 kV using an EMSIS Quemesa camera (11 Mpxl; Münster, Germany).

### 2.8 Bio-layer interferometry

First, hBACE1 (in-house produced ectodomain of human BACE1 protein) and hTfR1 (R&D systems, Minneapolis, United States; 2474-TR-050) were biotinylated with the EZ-Link NHS-PEG4-Biotinylation Kit (Thermo Fisher Scientific; 21455) according to the manufacturer instructions. Binding of HFn and modified HFn NPs to biotinylated hBACE1 and hTfR1 was assessed using Bio-layer interferometry (BLI) (Octet RED96 (Sartorius AG, Göttingen, Germany)). Briefly, streptavidin (SA) biosensor tips (Sartorius AG; 18-5019) were pre-wet for minimally 30 min in 1x Sartorius/Octet kinetic buffer, after which they were loaded with biotinylated target protein (5 μg/mL in 1x kinetic buffer) for 90 s, followed by a baseline step (1x kinetic buffer) for 120 s. The sensors were then moved to wells containing the HFn and modified HFn NPs (diluted in 1x kinetic buffer) for association and subsequently to wells with 1x kinetic buffer for dissociation (both phases 900 s). The raw data of real-time binding curves were generated using FortéBio Octet RED software and analyzed with GraphPad Prism 10 software.

### 2.9 hBACE1 and hTfR1 ELISA

A sandwich enzyme-linked immunosorbent assay (ELISA) format was used to screen for binding of modified HFn NPs. Briefly, 96-well plates (BD Falcon, Franklin Lakes, New Jersey, United States; 351172) were coated with 2 μg/mL purified hBACE1/hTfR1 ectodomain protein overnight at 4 °C. Following blocking with 0.1% casein (Sigma, St. Louis, Missouri, United States) in PBS for 2 h, the plates were incubated with the modified HFn NPs for 2 h at RT with gentle shaking. After washing, detection was performed by adding a mouse anti-human IgG Fc HRP-mAb (GenScript, Piscataway, NJ, United States; 50B4A9), diluted 1:5,000 in PBS containing 0.1% casein to detect 1A11-HFn, followed by incubation for 1 h at RT. After a final washing step, plates were developed by adding 100 μL of 1-step Ultra TMB substrate solution (Thermo Fisher Scientific; 34028). The enzymatic reaction was quenched by adding 2 N H_2_SO_4_, and the absorbance was immediately measured at 450 nm using a microplate reader.

### 2.10 Animals

As described previously, the genomic region encoding the mouse TfR apical domain (residues 196–381) was replaced with the homologous human sequence (residues 194–379), generating mice that express the chimeric hTfR (Wouters et al., 2022). hTfR-knock-in (KI) C57BL/6 mice were bred in-house, and both males and females (7–10 weeks old, 15–26 g) were used in this study. All mice were housed under specific pathogen-free conditions with a 12-h light/dark cycle and provided with standard rodent chow and water ad libitum, along with cotton nesting material. All animal procedures were approved by the KU Leuven Animal Ethics Committee (project P091/2022 and P213/2020) and were conducted in strict compliance with relevant governmental and European Union guidelines.

### 2.11 Intravenous injection

Intravenous (IV) injections were performed via the lateral tail vein. Prior to administration, mice were placed in a heating chamber at 40 °C for 10–15 min to induce vasodilation. Subsequently, the animals were secured in a restrainer, and all the NPs were injected at a fixed volume of 130 μL, with varying dilutions used to achieve the desired dosages.

### 2.12 Blood and brain collection

Treated hTfR-KI C57BL/6 mice were euthanized at designated time points via intraperitoneal (i.p.) injection of a lethal dose of pentobarbital (≥150 mg/kg; Dolethal^®^, Vetoquinol, Niel, Belgium). Blood was collected via cardiac puncture and transferred to K3-EDTA capillary blood collection tube (Sarstedt, Nümbrecht, Germany; NC9255927). Plasma was isolated by two sequential centrifugation steps (2,000 *g* for 10 min at 4 °C, followed by 16,000 *g* for 10 min at 4 °C) and stored at −20 °C. Following blood collection, brains were harvested after transcardial perfusion with heparinized PBS (1:1,000 dilution of 5,000 IU/mL; Leo Pharma, Ballerup, Denmark). Brains were hemisected, and the weight of each cerebral hemisphere was recorded before snap-freezing in liquid nitrogen, followed by storage at −80 °C until processing.

### 2.13 Quantification of Aβ_1–40_ using MSD immunoassay

Aβ_1–40_ levels in brain and plasma were quantified using an in-house Meso Scale Discovery (MSD) immunoassay as previously described by Cuypers et al. (2025). Briefly, brain hemispheres were homogenized in extraction buffer (0.4% diethylamine, 50 mM NaCl, Roche cOmplete protease inhibitors) using three 20 s cycles at 6 m/s with cooling intervals using a FastPrep-24 classic homogenizer (MP Biomedicals, Irvine, United States). Homogenates were ultracentrifuged 100,000 *g*, 1 h, 4 °C), and supernatants were neutralized with 0.5 M Tris–HCl (pH 6.8). MSD plates were coated overnight at 4 °C with capture antibody LTDA_Aβ_40_ (1.5 μg/mL) (Cuypers et al., 2025). Plates were washed with PBS-T (0.05% Tween 20) and blocked with 0.1% casein in PBS (4 h, RT, shaking at 700 rpm). Samples (plasma or 1:2 diluted brain homogenate) and standards (rodent Aβ_40_, rPeptide; 2.4–2,500 pg/mL) were mixed 1:2 with sulfoTAG-conjugated detection antibody (LTDA_rAβN) (Cuypers et al., 2025), incubated for 15 min at RT, added to the plates, and incubated overnight at 4 °C. Plates were washed, and signals were measured using an MSD reader with 2X Read Buffer. Concentrations were calculated via a 4-parameter logistic regression in GraphPad Prism 10.

### 2.14 Quantification of 1A11 and 1A11-HFn in brain and plasma

#### 2.14.1 Sample preparation

Brain samples were prepared by homogenizing each hemisphere in nine volumes of PBS containing 1% Igepal^®^ (Thermo Fisher Scientific; J61055-AP) and cOmplete™ protease inhibitor cocktail EDTA-free tablets (Roche Diagnostics, Mannheim, Germany; 5056489001). Homogenization was performed in Ceramic Bead Lysing Matrix D tubes (MP Biomedicals, Irvine, United States; 1169130-CF) using a FastPrep-24 homogenizer (MP Biomedicals, Irvine, United States) set to 6.5 m/s for two 45-s cycles, with a 1-min cooling interval on ice between cycles. The homogenates were briefly centrifuged (10,000 *g*, 5 min, 4 °C) and incubated on ice to remove foam, followed by a clarification spin (16,000 *g*, 10 min, 4 °C) to collect the supernatant. This extraction process was repeated twice on the resulting pellet to ensure maximal recovery.

#### 2.14.2 ELISA quantification

Nanoparticles concentrations in plasma and brain lysates were quantified using an in-house sandwich ELISA. High-binding plates were coated overnight at 4 °C with either 2 μg/mL mouse anti-human IgG Fc (GenScript, Piscataway, USA; V90401-1) for detection of 1A11 in brain lysates, or in-house hBACE1 for 1A11-HFn detection in both brain and plasma and 1A11 in plasma. After coating, plates were blocked with PBS containing 0.1% casein for 2 h at RT. Samples were diluted in blocking buffer and incubated on the plates for 2 h at RT with shaking (300 rpm). Quantification was performed using matrix-matched calibration curves (serial 2-fold dilutions in plasma or brain homogenate) starting at 14.5 ng/mL for 1A11 and 65.4 ng/mL for 1A11-HFn. Captured analytes were detected using HRP-conjugated antibodies diluted 1:5,000 in blocking buffer. Mouse anti-human IgG Fab (GenScript; A01855-200) was used for 1A11, while mouse anti-human IgG Fc (GenScript; A01854-200) was used for 1A11-HFn. All steps, except blocking, were preceded by washing with PBS containing 0.002% Tween 80. Signal development was carried out with 1-Step Ultra TMB-ELISA substrate (Thermo Fisher Scientific; 34029) for 30 min, stopped with 2 N H_2_SO_4_, and absorbance was measured immediately at 450 nm using a CLARIOstar Plus reader (BMG LABTECH, Ortenberg, Germany). Data analysis was performed by linear regression using GraphPad Prism 10.

### 2.15 Statistical analysis

Statistical analysis was performed using GraphPad Prism 10 (GraphPad Software, San Diego, United States). For PK data, an unpaired t-test with Welch’s correction was used to compare the concentrations of 1A11 and 1A11-HFn at Day 1. For PD data, differences in Aβ_1–40_ levels in plasma and brain across groups (PBS, HFn, 1A11, and 1A11-HFn NPs) were analyzed using a one-way ANOVA followed by Tukey’s multiple comparisons test. All data are presented as mean ± SD. Statistical significance is considered for a *p*-value < 0.05.

## 3 Results

### 3.1 Chemical conjugation and characterization of HFn and 1A11-HFn NPs

**FIGURE 2:**
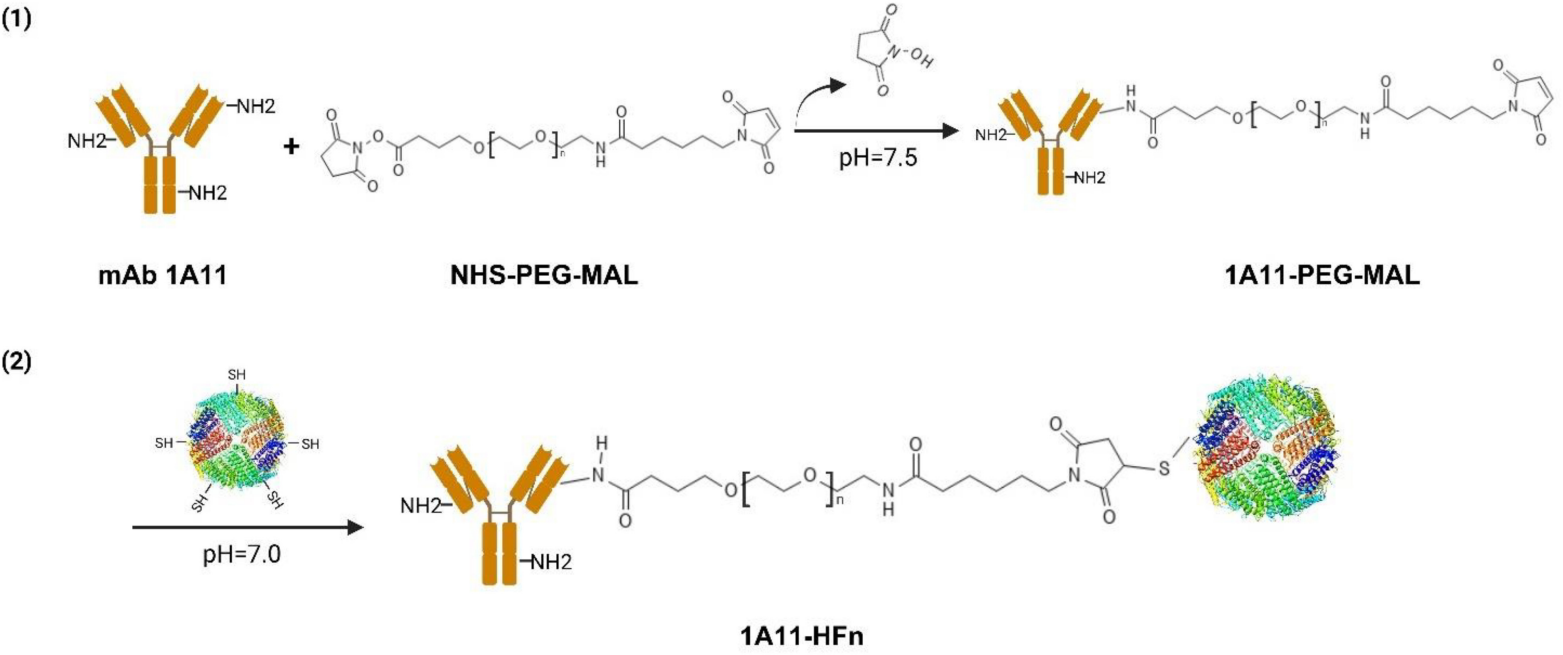
Generation of antibody-conjugated nanoparticles (NPs) based on H-ferritin (HFn) protein cages for hBACE1 inhibition. The HFn surface is chemically conjugated with mAb 1A11 (1A11-HFn) using a PEG-based heterobifunctional cross-linker (NHS-PEG-MAL).

H-ferritin NPs were produced in *E. coli* and purified by a multi-step purification protocol. After ammonium sulfate precipitation, endotoxins were removed by treatment with Triton X-114 followed by two consecutive size exclusion chromatography (SEC) steps. To form the 1A11-HFn NPs, an anti-BACE1 monoclonal antibody (mAb) 1A11 with a human IgG1 backbone was chemically coupled to the HFn surface. The presence of the human IgG backbone allows more straight forward detection of 1A11-HFn NPs in mouse samples. Coupling was achieved using a 5 kDa heterobifunctional PEG crosslinker containing N-hydroxysuccinimidyl ester (NHS) and maleimide (Mal) groups (Figure 2). Given the 1:1 ratio of 1A11:HFn NP used during the coupling, an average coupling of one mAb per HFn NP was assumed as molecular weight of 1A11-HFn NP from now on to calculate molarities, although in reality some NPs will most likely have no antibody fused while other will have more than one antibody. Such heterogeneity is inherently linked to chemical coupling reactions.

**FIGURE 3:**
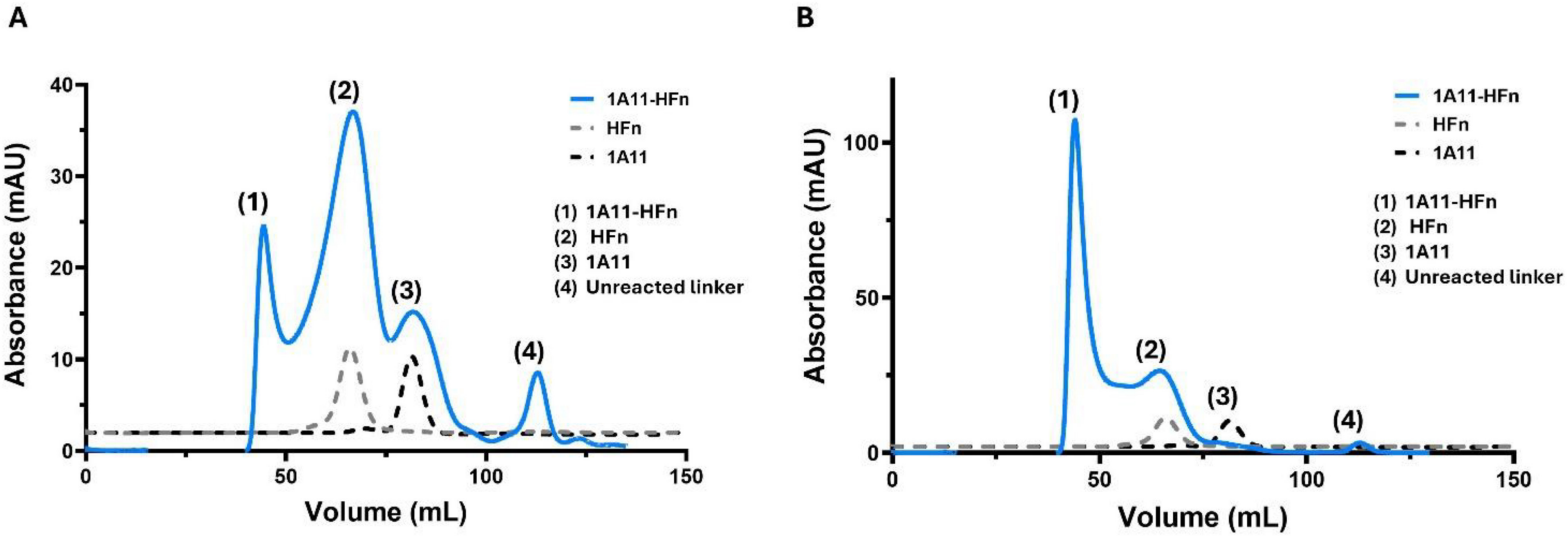
Size exclusion chromatography (SEC) characterization of 1A11-HFn NPs. SEC elution profiles of the nanoparticles (NPs) on a Superose 6 column following reactions performed at **(A)** low protein concentration and **(B)** high protein concentration.

Next, it was evaluated if the concentration of the proteins in the reaction influences the coupling efficiency. When the proteins were at a relatively low concentration in the coupling reaction (1 mg/ml), chemical conjugation was less efficient compared to higher protein concentrations during the coupling reaction (3–5 mg/mL). Indeed, the SEC profiles demonstrated that increasing the protein concentration during the reaction increased the yield of the target conjugate (Peak 1), confirming enhanced conjugation efficiency (Figures 3A,B). Based on analysis of the individual components by SEC and SDS-PAGE, Peak 1 corresponds to 1A11-HFn, Peak 2 to HFn, Peak 3 to 1A11 and Peak 4 to the unconjugated linker (Figures 3A,B and Supplementary Figure 1).

**FIGURE 4:**
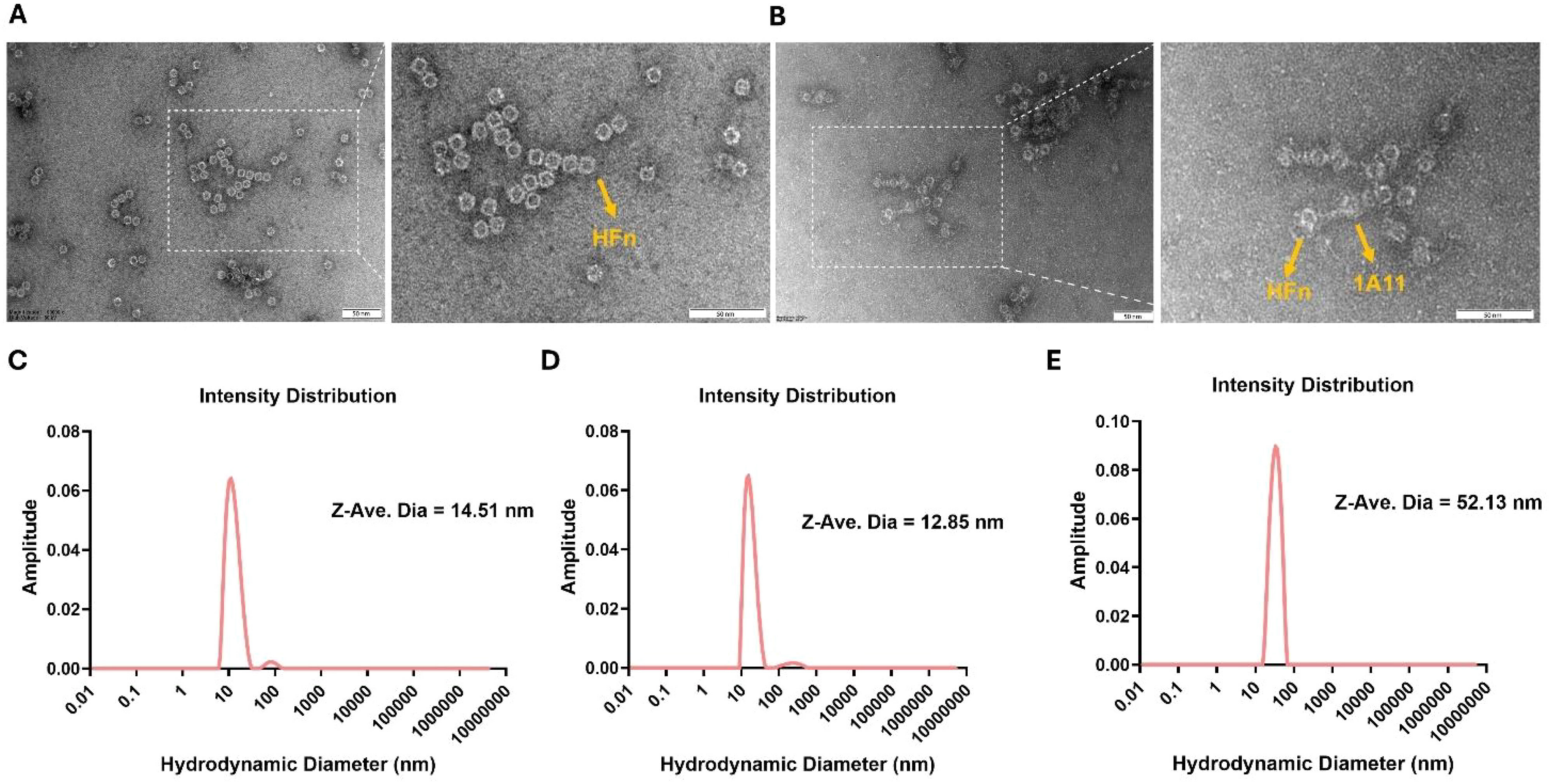
Nanoparticle morphology and size characterization. Representative transmission electron microscopy (TEM) images of H-ferritin (HFn) **(A)** and 1A11-HFn **(B)** NPs. Scale bars: 50 nm. DLS analysis of 1A11 **(C)**, HFn **(D)**, and 1A11-HFn **(E)**.

To confirm the integrity of HFn NPs after mAb 1A11 conjugation, the structural features of both negative-stained HFn NPs and 1A11-HFn NPs were analyzed using TEM. Structural analysis by TEM confirmed that the chemical modification preserved the characteristic hollow, spherical nanocage structure of HFn NPs (Figures 4A,B). Interestingly, for some of the observed NPs, additional density is detected, presumably originating from coupled antibodies (Figure 4B). In addition, DLS measurements confirmed the expected diameters for unconjugated HFn NPs and 1A11 [respectively, about 14.5 nm (Figure 4C) and 12.8 nm (Figure 4D)], while the final 1A11-HFn nanoconjugates exhibited an increased hydrodynamic size of approximately 50 nm (Figure 4E), which also suggests the successful formation of the 1A11-HFn NP complexes.

### 3.2 Binding affinity of 1A11-HFn NPs

**FIGURE 5:**
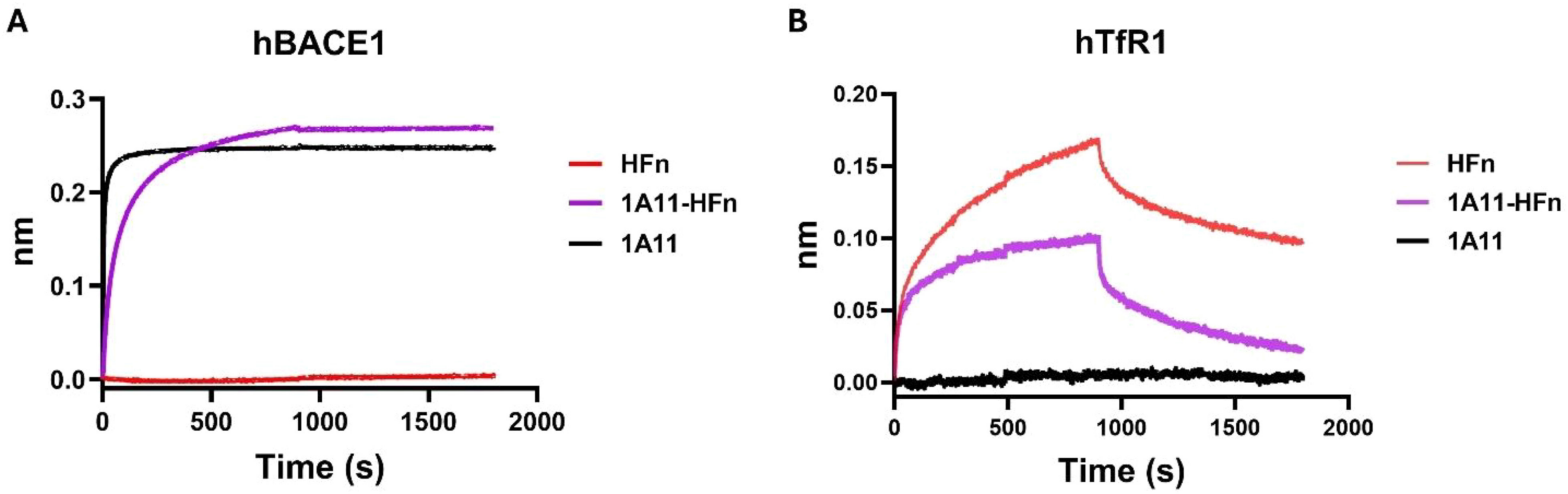
Bio-layer interferometry (BLI) analysis of 1A11-HFn binding to biotinylated hBACE1/hTfR1 immobilized on streptavidin biosensors. BLI was used to assess the binding of the 1A11–HFn (human IgG backbone, 1:1 reaction ratio), 1A11, and H-ferritin (HFn) to immobilized hBACE1 **(A)** and human TfR1 (hTfR1) **(B)**. Protein concentrations of 500 nM were used for hBACE1 binding and 250 nM for hTfR1 binding.

The purified 1A11-HFn NPs were further characterized *in vitro* for binding to their target receptors by BLI. Given the likely heterogeneity of the 1A11-HFn preparation (due to variations in the number of 1A11 molecules per HFn), accurate dissociation constant (KD) values could not be determined. Therefore, BLI was employed only to confirm that the preparations showed binding for their intended targets. As expected, all constructs containing HFn still demonstrated binding to hTfR, and all constructs containing mAb 1A11 still bound to hBACE1 (Figures 5A,B and Supplementary Figure 2). To confirm the observed BLI signal for 1A11-HFn is related to 1A11-HFn and not residual amounts of HFn still present in the samples, we also confirmed binding of 1A11-HFn to hTfR in an ELISA setup which specifically detects 1A11-HFn and not uncoupled HFn (Supplementary Figure 2).

### 3.3 *In vivo* PK/PD of 1A11-HFn NPs

**FIGURE 6:**
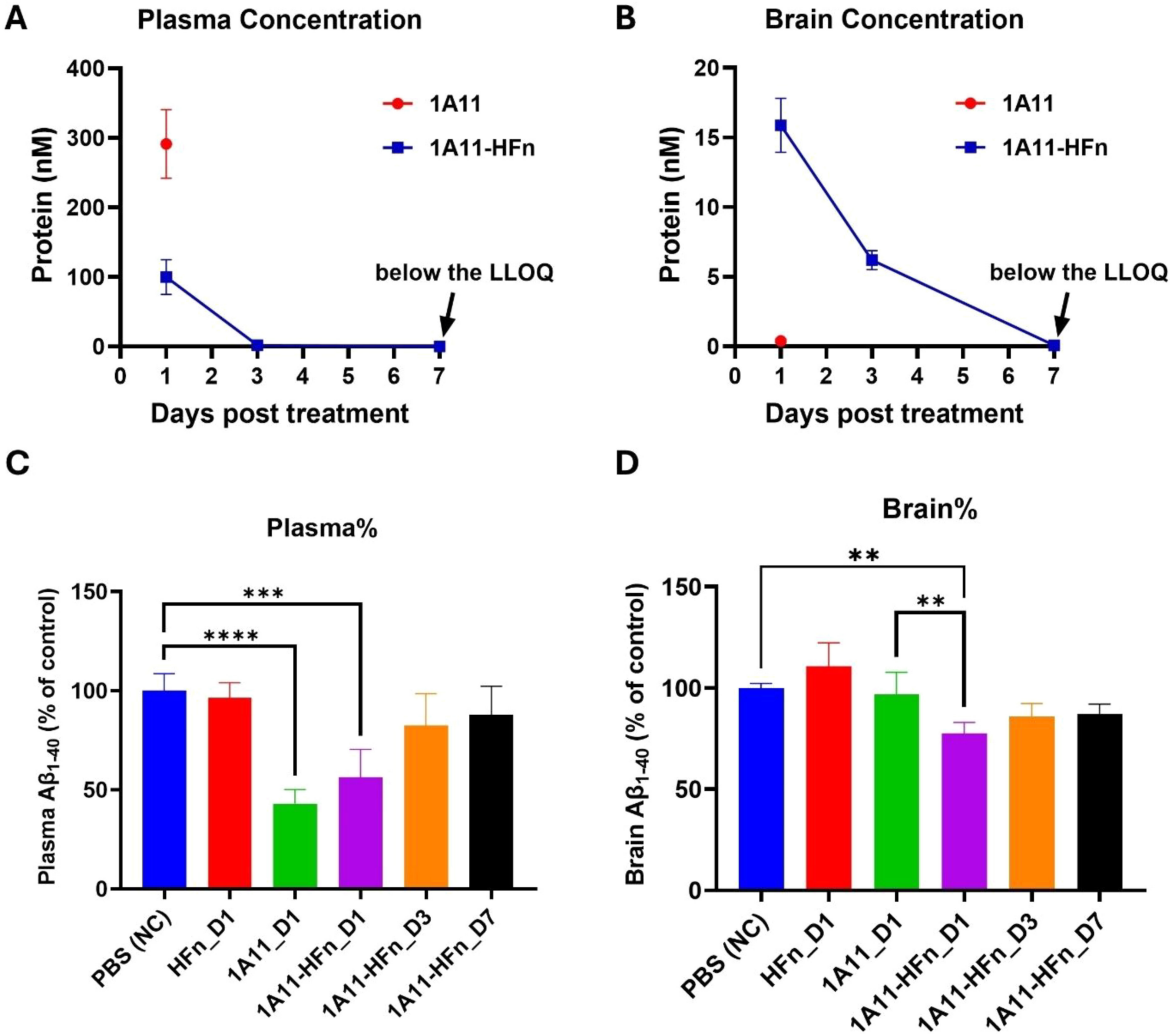
β_1–40_ levels and protein concentrations in plasma and brain of hTfR1-KI mice. Mice received a single intravenous injection (76.5 nmol/kg) of PBS (control), H-ferritin (HFn), 1A11, or 1A11-HFn. Pharmacokinetic (PK) profiles and pharmacodynamic (PD) effects were assessed at several timepoints up to 7 days. Plasma **(A)** and brain **(B)** concentration of treatment 1A11 and 1A11-HFn. Values represent mean ± SD (*n* = 6 per group). Effect of treatment on Aβ_1–40_ levels in plasma **(C)** and brain **(D)** displayed as % of control (average of all PBS treated mice of day 1). Bar graphs represent mean ± SD (*n* = 6 per group). Statistical analysis was performed using one-way ANOVA with Tukey’s multiple comparisons test to determine differences between all experimental groups for Aβ1-40 levels (**p < 0.01, ***p < 0.001, ****p < 0.0001). While the Figure highlights key significance between PBS and treatment groups (plasma) and between 1A11-HFn and other groups including PBS and unconjugated 1A11 (brain), comprehensive statistical data are provided in the Supplementary Table 1.

After the *in vitro* binding verification, the pharmacokinetic/ pharmacodynamic (PK/PD) profile of 1A11-HFn NPs was assessed *in vivo* in hTfR1-KI mice. The NPs and controls (uncoupled HFn and uncoupled 1A11) were administered via IV injection at a dose of 76.6 nmol/kg. Samples were collected at 1-, 3-, and 7-days post-treatment for the NPs and at day 1 for the controls. The PK profile was defined by determining the concentration of the 1A11-HFn NPs in plasma and brain at day 1, 3, and 7 and the concentration of control sample. 1A11 in plasma and brain was defined at day 1 (Figures 6A,B). 1A11-HFn NPs were detected in plasma 24 h post-treatment at 100 nM and an over 55-fold reduced concentration was still detected after 3 days. At day 7, plasma levels dropped below the lower limit of quantification (x̄_*blank*_ + 10σ; LLOQ = 0.008 nM). 1A11-HFn NPs were detected in the brain at ∼40-fold higher concentrations than unconjugated 1A11 (∼16 and 0.4 nM, respectively), indicating successful BBB-crossing of this construct. In contrast to plasma, the clearance from brain seems to be slower (with a 2.5-fold reduction observed by day 3 compared to day 1). At day 7, the 1A11-HFn was not detectable anymore in the brain (LLOQ = 0.04 nM).

Next, Aβ_1–40_ levels were determined in plasma and brain at the different timepoints. In plasma, both the unconjugated 1A11 and the 1A11-HFn NPs achieved a significant reduction in Aβ_1–40_ levels at day 1 compared to the PBS control but this effect was not observed at day 3 and 7 (Figure 6C). As expected, unconjugated 1A11 did not reduce Aβ_1–40_ levels in brain compared to the PBS control at the timepoint tested (day 1) (Figure 6D). In contrast, 1A11-HFn treatment resulted in decrease in Aβ_1–40_ levels at day 1 compared to unconjugated 1A11, but not at day 3 and 7, confirming that HFn can shuttle antibodies into the brain at pharmacologically relevant concentrations.

## 4 Discussion

Biologicals have revolutionized medicine in past decades, but so far have not delivered on their promise when it comes to CNS disorders (Pardridge, 2005). In terms of neurodegeneration, the medical need is urgent and continues to rise. Worldwide, an estimated 55 million people are affected by dementia (with Alzheimer’s disease (AD) as the most common cause), and more than 5 million by Parkinson’s disease (PD) (Georgescu et al., 2025). For all these diseases, but also other diseases such as cancer and autoimmune diseases like multiple sclerosis (MS), antibody therapy is under investigation, but delivery to the CNS is an important bottleneck (Terstappen et al., 2021). Under healthy conditions, the BBB precludes the free exchange of biologicals between blood and brain. While this barrier is of vital importance for brain health, it also severely hampers the delivery of drugs targeting CNS-related disorders (Pardridge, 2005). Immunotherapy trials for AD are most advanced, and recently Aβ-targeting mAbs (such as aducanumab, lecanemab) have been approved for clinical use (Sevigny et al., 2016; Chowdhury and Chowdhury, 2023). However, drug developers rely on the contention that very low CNS concentrations of these molecules (typically 0.1% of peripheral concentrations) will have a relevant pharmacodynamic effect in the brain. While this might be sufficient for some therapeutic antibodies, higher brain exposure might be required for others. Increasing the dose could be one potential solution to achieve higher brain exposure, but high peripheral doses increase the risk of peripheral side effects and treatment cost. Thus, even if successful to some extent, immunotherapy without active transport over the BBB will not be a final or long-term solution for the majority of neurological disorders. Therefore, in this study, we investigated whether HFn NPs, which naturally cross the BBB in a TfR-dependent manner, can also transport covalently coupled antibodies into the brain. To this end, we first conjugated an anti-BACE1 monoclonal antibody (1A11) to HFn via chemical linkage and thoroughly validated the resulting batches both *in vitro* and *in vivo*. After coupling, the batches were purified, and NP integrity was confirmed by TEM, while successful antibody conjugation was verified by DLS (increase in particle diameter after coupling). Binding to human TfR and BACE1 was also confirmed. We assessed the effect of 1A11-HFn NPs on plasma and brain Aβ_1–40_ levels at day 1, day 3 and day 7. It showed significant Aβ_1–40_ reduction, indicating that antibodies coupled to HFn can be delivered to the brain at pharmacologically relevant concentrations. This effect didn’t persist at later time points. The pharmacokinetic (PK) profile largely mirrored the pharmacodynamic (PD) observations: 1A11-HFn NPs were detected at elevated concentrations on days 1 and 3, but not day 7.

Closer examination revealed rapid NP clearance from plasma (>55-fold reduction between days 1 and 3; Figure 6A), consistent with the reported short intrinsic half-life of HFn (∼2–3 h in rodents) (Wang et al., 2018; Yin et al., 2021). In contrast, IgGs typically exhibit a long half-life (∼3 weeks) due to FcRn-mediated recycling (Roopenian and Akilesh, 2007), although target-mediated clearance often shortens this (An, 2020). Previous work, including ours, has shown that anti-BACE1 antibodies have relatively long half-lives, with only 2- to 3-fold reductions between days 1 and 3 post-injection (Yu et al., 2014; Wouters et al., 2022; Chew et al., 2023). This suggests that HFn-antibody conjugates adopt the half-life of HFn rather than the antibody. Interestingly, NP clearance from the brain seems to be slower than from plasma (approximately 2.5-fold reduction between days 1 and 3 in brain vs. >55-fold in plasma). Finally, although NP conjugates were still detectable in the brain at day 3 (∼6.2 nM), this concentration was insufficient for robust BACE1 inhibition.

Previously, we delivered anti-BACE1 mAb 1A11 to the brain using bispecific antibodies targeting both BACE1 and TfR (1A11-Nb62 and 1A11-Nb188) (Wouters et al., 2022; Rue et al., 2023). Nb62 and Nb188 bind to mouse and human TfR, respectively. For these constructs, we measured antibody and Aβ_1–40_ levels at days 1, 3, and 7 after injecting 167 nmol/kg, a dose ∼2-fold higher than the dose used for NP conjugates, warranting caution in comparisons. Nevertheless, bispecific antibodies achieved respectively around 7- and 5-fold higher brain concentrations at day 1 than NP conjugates, resulting in ∼50% Aβ_1–40_ reduction for both bispecific antibodies versus ∼20% for NP conjugates (±50% is the maximal Aβ_1–40_ reduction achievable with BACE1-inhibiting antibodies). Peripheral clearance of bispecific antibodies appears to be slower (4.2- and 11.5-fold reductions between days 1 and 3 vs. >55-fold for NP conjugates), but brain clearance appears to be faster (4.6- and 17.5-fold reductions vs. 2.5-fold for NP conjugates). Potential safety concerns have previously been reported for transferrin receptor–targeting antibodies used to shuttle cargo across the BBB, including an acute but transient reduction in reticulocyte counts (Couch et al., 2013). Abrogation of antibody effector function has been shown to mitigate this issue (Couch et al., 2013). Accordingly, the effector function of the antibody was eliminated in the present study. However, we did not assess reticulocyte levels, which represents a limitation of our work. Future studies evaluating HFn-based antibody shuttles should include systematic monitoring of reticulocyte counts to fully address this safety consideration.

Overall, these findings suggest that both HFn and TfR targeting antibodies can enable brain delivery of antibodies, but TfR targeting antibodies seem to be more efficient in doing this. In contrast, HFn conjugated antibodies seem to have a somewhat decreased brain clearance rate. Therefore, if someone just wants to delivery antibodies to the brain, it might be more straight forward and efficient to just make bispecific antibodies with an anti-TfR1 binding arm to shuttle it into the brain. However, NP conjugates may still offer additional advantages in specific applications not feasible with bispecific antibodies, such as targeted delivery of small-molecule drugs to the brain. As previously reported, Liang et al. demonstrated that HFn can encapsulate approximately 33 molecules of doxorubicin per NP using urea-mediated disassembly/reassembly method (Liang et al., 2014). This loading capacity was further improved through copper-mediated coordination (5-fold) (Zhen et al., 2013), while also high hydrostatic pressure increased loading by up to threefold compared with atmospheric conditions (Wang et al., 2017). Expanding beyond small chemical compounds, Yan et al. further bioengineered HFn to incorporate a positively charged internal cavity, generating HFn(+) variants that enable electrostatic encapsulation of TLR-activating nucleic acids, resulting in enhanced cellular uptake, lysosomal targeting, and immune activation (Zhang et al., 2022). Building on this design, the same group developed tHFn(+) variants with a truncated C-terminus, which further improved siRNA delivery and effectively suppressed gene expression associated with glioblastoma progression *in vivo* (Jin et al., 2025). Collectively, these findings highlight the remarkable versatility of HFn as a drug delivery platform. Compared with conventional antibody-drug conjugates (ADCs) and antibody-oligonucleotide conjugates (AOCs), HFn NPs offer substantially higher loading capacity and superior cargo protection by shielding therapeutic agents within a robust protein shell. Moreover, when combined with surface-conjugated antibodies that target specific brain regions or cell types, HFn-based systems could potentially enable the precise delivery of therapeutics across the BBB to desired sites within the brain.

## Data Availability

The original contributions presented in this study are included in this article/Supplementary material, further inquiries can be directed to the corresponding authors.

## References

[B1] Adelman T. G. Arosio P. Drysdale J. W. (1975). Multiple subunits in human ferritins: Evidence for hybrid molecules. *Biochem. Biophys. Res. Commun.* 63 1056–1062. 10.1016/0006-291x(75)90676-2 1131267

[B2] Alahmari A. (2021). Blood-brain barrier overview: Structural and functional correlation. *Neural Plast.* 2021:6564585. 10.1155/2021/6564585 34912450 PMC8668349

[B3] An G. (2020). Concept of pharmacologic target-mediated drug disposition in large-molecule and small-molecule compounds. *J. Clin. Pharmacol.* 60 149–163. 10.1002/jcph.1545 31793004 PMC7472685

[B4] An S. McInnis J. J. Kim D. Li Y. Tasdemir-Yilmaz O. Ahn J. et al. (2025). A brain-shuttled antibody targeting alpha synuclein aggregates for the treatment of synucleinopathies. *NPJ Parkinsons Dis.* 11:254. 10.1038/s41531-025-01117-6 40847026 PMC12373799

[B5] Blanco E. Shen H. Ferrari M. (2015). Principles of nanoparticle design for overcoming biological barriers to drug delivery. *Nat. Biotechnol.* 33 941–951. 10.1038/nbt.3330 26348965 PMC4978509

[B6] Boado R. J. Hui E. K. Lu J. Z. Pardridge W. M. (2016). Very high plasma concentrations of a monoclonal antibody against the human insulin receptor are produced by subcutaneous injection in the rhesus monkey. *Mol. Pharm.* 13 3241–3246. 10.1021/acs.molpharmaceut.6b00456 27513815

[B7] Cheng Y. Zak O. Aisen P. Harrison S. C. Walz T. (2004). Structure of the human transferrin receptor-transferrin complex. *Cell* 116 565–576. 10.1016/s0092-8674(04)00130-8 14980223

[B8] Chew K. S. Wells R. C. Moshkforoush A. Chan D. Lechtenberg K. J. Tran H. L. et al. (2023). CD98hc is a target for brain delivery of biotherapeutics. *Nat. Commun.* 14:5053. 10.1038/s41467-023-40681-4 37598178 PMC10439950

[B9] Chowdhury S. Chowdhury N. S. (2023). Novel anti-amyloid-beta (Abeta) monoclonal antibody lecanemab for Alzheimer’s disease: A systematic review. *Int. J. Immunopathol. Pharmacol.* 37:3946320231209839. 10.1177/03946320231209839 37902139 PMC10617290

[B10] Couch J. A. Yu Y. J. Zhang Y. Tarrant J. M. Fuji R. N. Meilandt W. J. et al. (2013). Addressing safety liabilities of TfR bispecific antibodies that cross the blood-brain barrier. *Sci. Transl. Med.* 5 181–112. 10.1126/scitranslmed.3005338 23636093

[B11] Cuypers M.-L. Jaspers T. Clerckx J. Leekens S. Cawthorne C. Bormans G. et al. (2025). Increasing brain half-life of antibodies by additional binding to myelin oligodendrocyte glycoprotein, a CNS specific protein. *Fluids Barriers CNS* 22:11. 10.1186/s12987-025-00624-1 39885527 PMC11783731

[B12] Fan K. Cao C. Pan Y. Lu D. Yang D. Feng J. et al. (2012). Magnetoferritin nanoparticles for targeting and visualizing tumour tissues. *Nat. Nanotechnol.* 7 459–464. 10.1038/nnano.2012.90 22706697

[B13] Fan K. Jia X. Zhou M. Wang K. Conde J. He J. et al. (2018). Ferritin nanocarrier traverses the blood brain barrier and kills glioma. *ACS Nano* 12 4105–4115. 10.1021/acsnano.7b06969 29608290

[B14] Farokhzad O. C. Langer R. (2009). Impact of nanotechnology on drug delivery. *ACS Nano* 3 16–20. 10.1021/nn900002m 19206243

[B15] Georgescu M. F. Beydoun M. A. Weiss J. Kubchandani J. Banerjee S. Gamaldo A. A. et al. (2025). Cardiovascular health and its association with dementia, Parkinson’s disease, and mortality among UK older adults. *Brain Behav. Immun. Health* 45:100986. 10.1016/j.bbih.2025.100986 40235832 PMC11999287

[B16] Giugliani R. Martins A. M. Okuyama T. Eto Y. Sakai N. Nakamura K. et al. (2021). Enzyme replacement therapy with pabinafusp alfa for neuronopathic mucopolysaccharidosis II: An integrated analysis of preclinical and clinical data. *Int. J. Mol. Sci.* 22:10938. 10.3390/ijms222010938 34681597 PMC8535651

[B17] Hampel H. Hardy J. Blennow K. Chen C. Perry G. Kim S. H. et al. (2021). The amyloid-beta pathway in Alzheimer’s disease. *Mol. Psychiatry* 26 5481–5503. 10.1038/s41380-021-01249-0 34456336 PMC8758495

[B18] Hersh A. M. Alomari S. Tyler B. M. (2022). Crossing the blood-brain barrier: Advances in nanoparticle technology for drug delivery in neuro-oncology. *Int. J. Mol. Sci.* 23:4153. 10.3390/ijms23084153 35456971 PMC9032478

[B19] Honarmand Ebrahimi K. Hagedoorn P. L. Hagen W. R. (2015). Unity in the biochemistry of the iron-storage proteins ferritin and bacterioferritin. *Chem. Rev.* 115 295–326. 10.1021/cr5004908 25418839

[B20] Jin Y. Zhang B. Li J. Guo Z. Zhang C. Chen X. et al. (2025). Bioengineered protein nanocarrier facilitating siRNA escape from lysosomes for targeted RNAi therapy in glioblastoma. *Sci. Adv.* 11:eadr9266. 10.1126/sciadv.adr9266 39970222 PMC11838010

[B21] Koralewski M. Balejcikova L. Mitroova Z. Pochylski M. Baranowski M. Kopcansky P. (2018). Morphology and magnetic structure of the ferritin core during iron loading and release by magnetooptical and NMR methods. *ACS Appl. Mater. Interfaces* 10 7777–7787. 10.1021/acsami.7b18304 29417811

[B22] Li L. Fang C. J. Ryan J. C. Niemi E. C. Lebron J. A. Bjorkman P. J. et al. (2010). Binding and uptake of H-ferritin are mediated by human transferrin receptor-1. *Proc. Natl. Acad. Sci. U S A.* 107 3505–3510. 10.1073/pnas.0913192107 20133674 PMC2840523

[B23] Liang M. Fan K. Zhou M. Duan D. Zheng J. Yang D. et al. (2014). H-ferritin-nanocaged doxorubicin nanoparticles specifically target and kill tumors with a single-dose injection. *Proc. Natl. Acad. Sci. U S A.* 111 14900–14905. 10.1073/pnas.1407808111 25267615 PMC4205604

[B24] Lin X. Koelsch G. Wu S. Downs D. Dashti A. Tang J. (2000). Human aspartic protease memapsin 2 cleaves the beta-secretase site of beta-amyloid precursor protein. *Proc. Natl. Acad. Sci. U S A.* 97 1456–1460. 10.1073/pnas.97.4.1456 10677483 PMC26455

[B25] Liu W. Lin Q. Fu Y. Huang S. Guo C. Li L. et al. (2020). Target delivering paclitaxel by ferritin heavy chain nanocages for glioma treatment. *J. Control Release* 323 191–202. 10.1016/j.jconrel.2019.12.010 31838201

[B26] Lu R. M. Hwang Y. C. Liu I. J. Lee C. C. Tsai H. Z. Li H. J. et al. (2020). Development of therapeutic antibodies for the treatment of diseases. *J. Biomed. Sci.* 27:1. 10.1186/s12929-019-0592-z 31894001 PMC6939334

[B27] Lyu X. Zhao Q. Hui J. Wang T. Lin M. Wang K. et al. (2022). The global landscape of approved antibody therapies. *Antib. Ther.* 5 233–257. 10.1093/abt/tbac021 36213257 PMC9535261

[B28] Mancias J. D. Wang X. Gygi S. P. Harper J. W. Kimmelman A. C. (2014). Quantitative proteomics identifies NCOA4 as the cargo receptor mediating ferritinophagy. *Nature* 509 105–109. 10.1038/nature13148 24695223 PMC4180099

[B29] Masserini M. (2013). Nanoparticles for brain drug delivery. *ISRN Biochem.* 2013:238428. 10.1155/2013/238428 25937958 PMC4392984

[B30] Mitchell M. J. Billingsley M. M. Haley R. M. Wechsler M. E. Peppas N. A. Langer R. (2021). Engineering precision nanoparticles for drug delivery. *Nat. Rev. Drug Discov.* 20 101–124. 10.1038/s41573-020-0090-8 33277608 PMC7717100

[B31] Mitragotri S. Burke P. A. Langer R. (2014). Overcoming the challenges in administering biopharmaceuticals: Formulation and delivery strategies. *Nat. Rev. Drug Discov.* 13 655–672. 10.1038/nrd4363 25103255 PMC4455970

[B32] Montemiglio L. C. Testi C. Ceci P. Falvo E. Pitea M. Savino C. et al. (2019). Cryo-EM structure of the human ferritin-transferrin receptor 1 complex. *Nat. Commun.* 10:1121. 10.1038/s41467-019-09098-w 30850661 PMC6408514

[B33] Pardridge W. M. (2005). The blood-brain barrier: Bottleneck in brain drug development. *NeuroRx* 2 3–14. 10.1602/neurorx.2.1.3 15717053 PMC539316

[B34] Pardridge W. M. (2019). Blood-brain barrier and delivery of protein and gene therapeutics to brain. *Front. Aging Neurosci.* 11:373. 10.3389/fnagi.2019.00373 31998120 PMC6966240

[B35] Patra J. K. Das G. Fraceto L. F. Campos E. V. R. Rodriguez-Torres M. D. P. Acosta-Torres L. S. et al. (2018). Nano based drug delivery systems: Recent developments and future prospects. *J. Nanobiotechnol.* 16:71. 10.1186/s12951-018-0392-8 30231877 PMC6145203

[B36] Preston J. E. Joan Abbott N. Begley D. J. (2014). Transcytosis of macromolecules at the blood-brain barrier. *Adv. Pharmacol.* 71 147–163. 10.1016/bs.apha.2014.06.001 25307216

[B37] Rathi S. Griffith J. I. Zhang W. Zhang W. Oh J. H. Talele S. et al. (2022). The influence of the blood-brain barrier in the treatment of brain tumours. *J. Intern. Med.* 292 3–30. 10.1111/joim.13440 35040235

[B38] Rizzuto M. A. Dal Magro R. Barbieri L. Pandolfi L. Sguazzini-Viscontini A. Truffi M. et al. (2021). H-Ferritin nanoparticle-mediated delivery of antibodies across a BBB in vitro model for treatment of brain malignancies. *Biomater. Sci.* 9 2032–2042. 10.1039/d0bm01726d 33544109

[B39] Roopenian D. C. Akilesh S. (2007). FcRn: The neonatal Fc receptor comes of age. *Nat. Rev. Immunol.* 7 715–725. 10.1038/nri2155 17703228

[B40] Rue L. Jaspers T. Degors I. M. S. Noppen S. Schols D. De Strooper B. et al. (2023). Novel human/non-human primate cross-reactive anti-transferrin receptor nanobodies for brain delivery of biologics. *Pharmaceutics* 15:1748. 10.3390/pharmaceutics15061748 37376196 PMC10300862

[B41] Sevieri M. Mazzucchelli S. Barbieri L. Garbujo S. Carelli S. Bonizzi A. et al. (2023). Ferritin nanoconjugates guide trastuzumab brain delivery to promote an antitumor response in murine HER2 + breast cancer brain metastasis. *Pharmacol. Res.* 196:106934. 10.1016/j.phrs.2023.106934 37734460

[B42] Sevigny J. Chiao P. Bussiere T. Weinreb P. H. Williams L. Maier M. et al. (2016). The antibody aducanumab reduces Abeta plaques in Alzheimer’s disease. *Nature* 537 50–56. 10.1038/nature19323 27582220

[B43] Shi J. Kantoff P. W. Wooster R. Farokhzad O. C. (2017). Cancer nanomedicine: Progress, challenges and opportunities. *Nat. Rev. Cancer* 17 20–37. 10.1038/nrc.2016.108 27834398 PMC5575742

[B44] Sitia L. Bonizzi A. Mazzucchelli S. Negri S. Sottani C. Grignani E. et al. (2021). Selective targeting of cancer-associated fibroblasts by engineered H-ferritin nanocages loaded with navitoclax. *Cells* 10:328. 10.3390/cells10020328 33562504 PMC7915356

[B45] Terstappen G. C. Meyer A. H. Bell R. D. Zhang W. (2021). Strategies for delivering therapeutics across the blood-brain barrier. *Nat. Rev. Drug Discov.* 20 362–383. 10.1038/s41573-021-00139-y 33649582

[B46] Tetter S. Hilvert D. (2017). Enzyme encapsulation by a ferritin cage. *Angew. Chem. Int. Ed. Engl.* 56 14933–14936. 10.1002/anie.201708530 28902449

[B47] Torchilin V. P. (2014). Multifunctional, stimuli-sensitive nanoparticulate systems for drug delivery. *Nat. Rev. Drug Discov.* 13 813–827. 10.1038/nrd4333 25287120 PMC4489143

[B48] Wang B. Tang M. Yuan Z. Li Z. Hu B. Bai X. et al. (2022). Targeted delivery of a STING agonist to brain tumors using bioengineered protein nanoparticles for enhanced immunotherapy. *Bioact. Mater.* 16 232–248. 10.1016/j.bioactmat.2022.02.026 35386310 PMC8965725

[B49] Wang C. Zhang C. Li Z. Yin S. Wang Q. Guo F. et al. (2018). Extending half life of H-ferritin nanoparticle by fusing albumin binding domain for doxorubicin encapsulation. *Biomacromolecules* 19 773–781. 10.1021/acs.biomac.7b01545 29328653

[B50] Wang Q. Zhang C. Liu L. Li Z. Guo F. Li X. et al. (2017). High hydrostatic pressure encapsulation of doxorubicin in ferritin nanocages with enhanced efficiency. *J. Biotechnol.* 254 34–42. 10.1016/j.jbiotec.2017.05.025 28591619

[B51] Wang Y. Gao S. Ye W.-H. Yoon H. S. Yang Y.-Y. (2006). Co-delivery of drugs and DNA from cationic core–shell nanoparticles self-assembled from a biodegradable copolymer. *Nat. Mater.* 5 791–796. 10.1038/nmat1737 16998471

[B52] Wouters Y. Jaspers T. Rue L. Serneels L. De Strooper B. Dewilde M. (2022). VHHs as tools for therapeutic protein delivery to the central nervous system. *Fluids Barriers CNS* 19:79. 10.1186/s12987-022-00374-4 36192747 PMC9531356

[B53] Yin S. Wang Y. Zhang B. Qu Y. Liu Y. Dai S. et al. (2021). Engineered human heavy-chain ferritin with half-life extension and tumor targeting by PAS and RGDK peptide functionalization. *Pharmaceutics* 13:521. 10.3390/pharmaceutics13040521 33918853 PMC8070472

[B54] Yu Y. J. Atwal J. K. Zhang Y. Tong R. K. Wildsmith K. R. Tan C. et al. (2014). Therapeutic bispecific antibodies cross the blood-brain barrier in nonhuman primates. *Sci. Transl. Med.* 6:261ra154. 10.1126/scitranslmed.3009835 25378646

[B55] Zhang B. Chen X. Tang G. Zhang R. Li J. Sun G. et al. (2022). Constructing a nanocage-based universal carrier for delivering TLR-activating nucleic acids to enhance antitumor immunotherapy. *Nano Today* 46:101564. 10.1016/j.nantod.2022.101564

[B56] Zhang S. Jin Y. Hou Y. Tang G. Wang Z. Chen X. et al. (2026). Bioengineered ferritin-based lysosome-targeting chimera platform for tumor-targeted therapy. *Nat. Commun.* 17:3706. 10.1038/s41467-026-70383-6 41803113 PMC13102911

[B57] Zhao P. Zhang N. An Z. (2022). Engineering antibody and protein therapeutics to cross the blood-brain barrier. *Antib. Ther.* 5 311–331. 10.1093/abt/tbac028 36540309 PMC9759110

[B58] Zhen Z. Tang W. Chen H. Lin X. Todd T. Wang G. et al. (2013). RGD-modified apoferritin nanoparticles for efficient drug delivery to tumors. *ACS Nano* 7 4830–4837. 10.1021/nn305791q 23718215 PMC3705644

[B59] Zuchero Y. J. Chen X. Bien-Ly N. Bumbaca D. Tong R. K. Gao X. et al. (2016). Discovery of novel blood-brain barrier targets to enhance brain uptake of therapeutic antibodies. *Neuron* 89 70–82. 10.1016/j.neuron.2015.11.024 26687840

