# Supplementary material for: H-ferritin nanoparticle-mediated antibody delivery across the blood-brain barrier
Source: Front Aging Neurosci. 2026 Jun 3;18:1839772. doi: 10.3389/fnagi.2026.1839772 (PMC13272470; doi:10.3389/fnagi.2026.1839772)
Supplement: Supplementary file 1 [file Data_Sheet_1.docx]

**Supplementary Information**

**H-ferritin nanoparticle-mediated antibody delivery across the blood-brain barrier**

Ziwei Yuan^1^, Laura Rué^1^, Tom Jaspers^1^, Marie-Lynn Cuypers^1^, and Maarten Dewilde^1,2,*^

1. Laboratory for Therapeutic and Diagnostic Antibodies, KU Leuven - University of Leuven, O&N II Herestraat 49 box 820, 3000, Leuven, Belgium
2. PharmAbs - the KU Leuven Antibody Center, KU Leuven - University of Leuven, O&N II Herestraat 49 box 820, 3000, Leuven, Belgium

* Corresponding author: maarten.dewilde@kuleuven.be (M.D.)


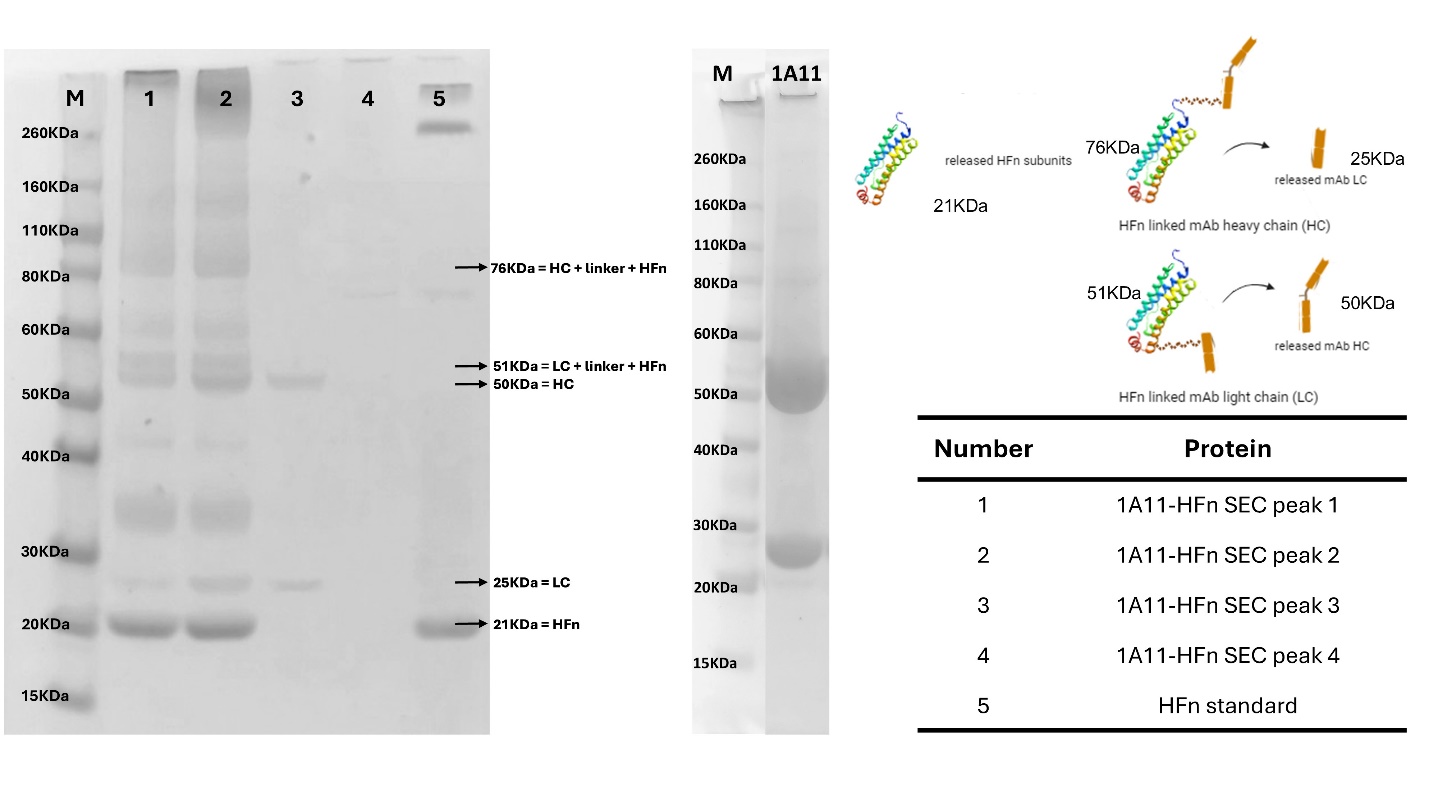


**Supplementary Figure 1. SDS-PAGE analysis of 1A11-HFn NPs.** Schematic representation of subunit release mechanism following reducing SDS-PAGE and SDS-PAGE results for the generated 1A11-HFn NPs.


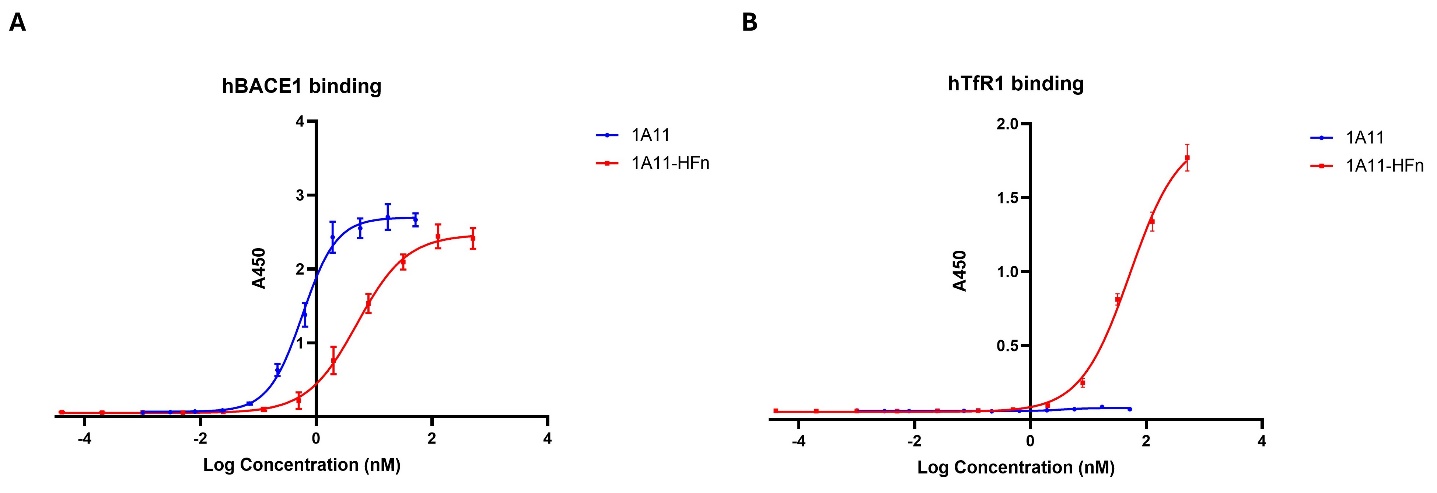


**Supplementary Figure 2. ELISA binding to hBACE1 and hTfR1.** ELISA assessment of the binding activity of 1A11 and the 1A11-HFn NPs to hBACE1 **(A)** and hTfR1 **(B)**.

| **Figure/Segment** | **Statistical Test** | **Groups** | **α level** | **P value** |
| --- | --- | --- | --- | --- |
| Figure 6A (plasma) | Unpaired t  test with Welch’s correction | 1A11 vs. 1A11-HFn D1 | 0.05 | 0.0019 |
| Figure 6B (brain) | Unpaired t  test with Welch’s correction | 1A11 vs. 1A11-HFn D1 | 0.05 | <0.0001 |
| Figure 6C (plasma) | One-way ANOVA | PBS (NC) vs. HFn_D1 | 0.05 | 0.9988 |
|  |  | PBS (NC) vs. 1A11_D1 | 0.05 | <0.0001 |
|  |  | PBS (NC) vs. 1A11-HFn_D1 | 0.05 | 0.0009 |
|  |  | PBS (NC) vs. 1A11-HFn_D3 | 0.05 | 0.4093 |
|  |  | PBS (NC) vs. 1A11-HFn_D7 | 0.05 | 0.7551 |
|  |  | HFn_D1 vs. 1A11_D1 | 0.05 | <0.0001 |
|  |  | HFn_D1 vs. 1A11-HFn_D1 | 0.05 | 0.0009 |
|  |  | HFn_D1 vs. 1A11-HFn_D3 | 0.05 | 0.5663 |
|  |  | HFn_D1 vs. 1A11-HFn_D7 | 0.05 | 0.9036 |
|  |  | 1A11_D1 vs. 1A11-HFn_D1 | 0.05 | 0.6126 |
|  |  | 1A11_D1 vs. 1A11-HFn_D3 | 0.05 | 0.0011 |
|  |  | 1A11_D1 vs. 1A11-HFn_D7 | 0.05 | 0.0002 |
|  |  | 1A11-HFn_D1 vs. 1A11-HFn_D3 | 0.05 | 0.0190 |
|  |  | 1A11-HFn_D1 vs. 1A11-HFn_D7 | 0.05 | 0.0036 |
|  |  | 1A11-HFn_D3 vs. 1A11-HFn_D7 | 0.05 | 0.9784 |
| Figure 6D (brain) | One-way ANOVA | PBS (NC) vs. HFn_D1 | 0.05 | 0.2109 |
|  |  | PBS (NC) vs. 1A11_D1 | 0.05 | 0.9561 |
|  |  | PBS (NC) vs. 1A11-HFn_D1 | 0.05 | 0.0011 |
|  |  | PBS (NC) vs. 1A11-HFn_D3 | 0.05 | 0.0426 |
|  |  | PBS (NC) vs. 1A11-HFn_D7 | 0.05 | 0.0693 |
|  |  | HFn_D1 vs. 1A11_D1 | 0.05 | 0.1229 |
|  |  | HFn_D1 vs. 1A11-HFn_D1 | 0.05 | <0.0001 |
|  |  | HFn_D1 vs. 1A11-HFn_D3 | 0.05 | 0.0003 |
|  |  | HFn_D1 vs. 1A11-HFn_D7 | 0.05 | 0.0006 |
|  |  | 1A11_D1 vs. 1A11-HFn_D1 | 0.05 | 0.0061 |
|  |  | 1A11_D1 vs. 1A11-HFn_D3 | 0.05 | 0.2269 |
|  |  | 1A11_D1 vs. 1A11-HFn_D7 | 0.05 | 0.3405 |
|  |  | 1A11-HFn_D1 vs. 1A11-HFn_D3 | 0.05 | 0.4196 |
|  |  | 1A11-HFn_D1 vs. 1A11-HFn_D7 | 0.05 | 0.2755 |
|  |  | 1A11-HFn_D3 vs. 1A11-HFn_D7 | 0.05 | 0.9997 |

**Supplementary Table 1. Other statistical comparisons.**
